# Acknowledgment to Reviewers of *Biomedicines* in 2020

**DOI:** 10.3390/biomedicines9020105

**Published:** 2021-01-22

**Authors:** Biomedicines Editorial Office

**Affiliations:** MDPI AG, St. Alban-Anlage 66, 4052 Basel, Switzerland

Peer review is the driving force of journal development, and reviewers are gatekeepers who ensure that *Biomedicines* maintains its standards for the high quality of its published papers. Thanks to the cooperation of our reviewers, in 2020, the median time to first decision was 15 days and the median time to publication was 34 days. The editors would like to express their sincere gratitude to the following reviewers for their precious time and dedication, regardless of whether the papers were finally published:
Abd El Hakim, YasminaLu, PaulAbugri, Daniel A.Lu, Yong-ChenAcheva, AnnaLuchini, ClaudioAdamson, David CoryLuciani, MarcoAdamus, GrazynaLuckenbach, TillAdhikari, ManishLukyanov, Pavel A.Adly, Frady G.Lundstrom, KennethAdonakis, GeorgeLunt, PeterAfrin, SadiaLuzi, LivioAggas, JohnMa, BuyongAguilera-Correa, John JairoMa, Dik-LungAguirre, AitorMa, Zheng FeeiAhn, JiyeonMaciejczyk, MateuszAkagi, SatoshiMaçôas, ErmelindaAkita, SadanoriMadonov, PavelAl-Abdullah, Ismail H.Maghin, EdoardoAlaimo, AlessandroMagkoev, Tamerlan T.Alam, Mohammad ParvezMahli, AbdoAlbano, FrancescoMahmoud, AymanAlbarracín, Sonia L.Maibach, Howard I.Albu, SilviuMaioli, SilviaAldamiz-Echevarría, LuisMajkowska-Pilip, AgnieszkaAlessenko, Alice V.Majoor-Krakauer, DanielleAlessio, GambinoMajtan, TomasAlexander, Brenda M.Malapelle, UmbertoAlfarouk, Khalid OmerMalejczyk, JacekAl-Hilaly, YoussraMalfitano, Anna MariaAl-Khrasani, MahmoudMałgorzewicz, SylwiaAllen, ClaireMallis, PanagiotisAlleva, DavidMammadova-Bach, ElminaAllione, AlessandraManchia, MirkoAlonso, FranciscoMancinelli, RominaAlpar, DonatMandala, AshokAltenburg, ArwenManfredi, FrancescoAltintas, Mehmet M.Mannello, FerdinandoAltomonte, JenniferMänner, JörgAlvarez-Malmagro, JuliaMånsson, SvenAlves, MarcoMansuri, ShahidAmantini, ConsueloMantamadiotis, TheoAmarpuri, GauravMantuano, ElideAmelio, IvanoMarchuk, Douglas A.Amiral, JeanMardešić-Brakus, SnježanaAmoako, Akwasi AtakoraMargulis, BorisAmornyotin, SomchaiMarimpietri, DaniloAndereggen, LukasMarkkanen, EnniAndrade-Cetto, AdolfoMarkworth, James F.Angelini, PaolaMarnetto, FabianaAngrand, Pierre-OlivierMaroni, PaolaAntognelli, CinziaMarques, TaniaAnton, LennikovMartens, GerardAragonés, JuliánMartinez Calejman, CamilaAran, GemmaMartínez, Carlos M.Araniciu, CătălinMartinotti, SimonaArcucci, AlessandroMartu, Maria AlexandraArif, TasleemMaruyama, KeiAriyoshi, WataruMasalova, Olga V.Arlt, AlexanderMasarone, DanieleArnaud, FrançoiseMascitti, MarcoArroba, Ana I.Maspero, CinziaArsenijevic, TatjanaMassa, AnnamariaArtín-Aragón, Sagrario M.Masthoff, MaxAsano, RyutaroMastinu, AndreaAseervatham, JayaMastrotto, FrancescaAshihara, EishiMasumoto, HidetoshiAshour, HossamMata, ManuelAsimakopoulos, AnastasiaMatowicka-Karna, JoannaAsnicar, FrancescoMatsuda, AkioAspatwar, AshokMatsukawa, NoriyukiAspriello, Simone DomenicoMatsunaga, KazuhisaAtienza-Mateo, BelénMatsunaga, YasukaAvdonin, Pavel V.Matsuo, MasafumiAvtanski, DimiterMatte, AlessandroAwatade, Nikhil T.Mattioli-Belmonte, MonicaAyon, RamonMaugeri, Andrea GiuseppeAyoub, NassimMauri, EmanueleBabickova, JankaMauro, AnnunziataBacakova, LucieMavridis, KonstantinosBacci, StefanoMavropoulos, AthanasiosBąchor, RemigiuszMawhinney, ThomasBadimon, Juan J.Mayne, ChristopherBae, Ji-YeongMazur-Bialy, AgnieszkaBaek, Kwang-HyunMcClelland, GrantBahji, AneesMccully, Kilmer S.Baietti, Maria FrancescaMcLachlan, StelaBailey, Charles G.McMahon, MeaganBailly, ChristianMcMartin, KennethBaker, Mark D.Mcmorrow, TaraBakht, MartinMeccariello, RosariaBakšytė, GiedreMeer, MargaritaBalas, MihaelaMeerson, AriBaldi, AlfonsoMehn, DoraBalmer, LoisMehta, Gaurav A.Baluta, SylwiaMeijer, OnnoBalzano, TizianoMeléndez-Hevia, EnriqueBandapalli, Obul ReddyMelo, Bernardete F.Banerjee, JaideepMéndez-Barbero, NereaBanerjee, KalpitaMenke, Aswin L.Baniene, RasaMentis, Alexios Fotios A.Banskota, Arjun H.Merchán, MIguel ÁngelBanti, Christina N.Merlino, FrancescoBarabutis, NektariosMesquita, JoãoBarahona, IsabelMetsälä, MarkusBaran, JaroslawMetyas, SamyBarilani, MarioMetzinger, LaurentBarone, PaulMeurman, JukkaBar-Or, DavidMiao, LinglingBarr, Kelli L.Micale, NicolaBartoszewski, RafalMiceli, VitaleBartuzi, DamianMicha, DimitraBasak, SujitMichishita, RyomaBastola, PrabhakarMieliauskaitė, DianaBastrzyk, AnnaMiere, AlexandraBattistini, LuciaMiller, Robert H.Battula, NarendraMiltyk, WojciechBaudis, StefanMin, Kyung-SanBaysan, AylinMinamisawa, SusumuBazil, JasonMinh, TruongngocBazylińska, UrszulaMiniero, Daniela ValeriaBeberok, ArturMinucci, SergioBeckingham, BryanMiotto, BenoitBehling, FelixMishra, AbheepsaBehringer, ErikMistiaen, WilhelmBeis, DimitrisMita, KojiBellei, BarbaraMitraki, AnnaBello, Nicholas T.Mitsios, AlexandrosBelosludtsev, KonstantinMittal, NiteshBenninghoff, AbbyMittempergher, LorenzaBerardo, ClarissaMizukawa, HazukiBeretta, Giovanni L.Mleczko-Sanecka, KatarzynaBereza-Malcolm, LaraModahl, CassandraBernardi, SimonaMoghimi, Seyed MoeinBertoncello, PaoloMohr, AndreaBesharat, Zein MersiniMohr, ThomasBessette, BarbaraMok, Daniel Kam-WahBessho, TadayoshiMoldzio, RudolfBest, JanMolina-Cerrillo, JavierBeszédes, SándorMoloney, Gerard M.Bezu, LucilliaMondal, GoutamBhardwaj, GouravMondal, SudipBhargava, AditiMonga, VarunBhatti, Fazal-Ur-RehmanMonteiro, LuisBiagini, GiuseppeMonteiro, SilviaBienboire-Frosini, CecileMoonen, Carolyn G.J.Billerbeck, SonjaMoraca, FedericaBilski, JanMorales, PaulaBirrell, MarkMorbidelli, LuciaBitto, AlessandraMorciano, GiampaoloBlasiak, JanuszMorcillo, Miguel ÁngelBlot-Chabaud, MarcelMorgan, EthanBoaventura, PaulaMorgan-Bathke, MariaBobis-Wozowicz, SylwiaMori, KiyoshiBoda, FranciscMorimura, ShigeruBoehme, Karen A.Morini, MartinaBoekema, BoukeMorouço, PedroBoerma, MarjanMosqueira, MatiasBogomolovas, JulijusMotlík, JanBohlmann, MichaelMousseau, DarrellBoldorini, RenzoMouzaki, AthanasiaBonam, Srinivasa ReddyMoyá, María LuisaBonaventura, AldoMueller, PhilippBonnländer, BerndMueller, TonyBontempo, PaolaMuga, ArturoBooth, BrianMühle, ChristianeBorcherding, Nicholas C.Mukaida, NaofumiBose, DebojitMukherjee, RajibBoso, DanieleMukherjee, SudipBosse, Jens B.Mukundan, SanthoshBoström, Jan PatrickMüller, ChristianBougea, AnastasiaMuller, Patricia A. J.Bourneuf, EmmanuelleMuntane, JordiBousiges, OlivierMuntean, Alexandru AndreiBouvier, SylvieMuramatsu, TakashiBovolin, PatriziaMurase, DaikiBowling, Heather L.Murdaca, GiuseppeBozic, JoskoMustachio, LisaBraconi, DanielaMutlu, Ayse SenaBradshaw, NicholasMutreja, IshaBrady, Graham F.Mutsaers, HenricusBrai, AnnalauraMutti, LucianoBraicu, CorneliaMutus, BulentBranca, Jacopo Junio ValerioMysliveček, JaromírBrandt, Maarten M.Myung, WoojaeBraun, ThorstenNadǎş, George CosminBraunschweig, TillNaderi, MohammadBravo, Susana B.Nagano, SeiichiBrennan, GregNagaraj, Ram H.Bright, Fiona M.Nagaraj, ViniBrignole, ChiaraNagy, Előd ErnőBrinson, RobertNahalkova, JarmilaBrodosi, LuciaNahomi, Rooban B.Broering, RuthNair, Praful R.Brovkina, OlgaNakamura, NobuhiroBrown, Christopher LyonNakano, KeisukeBrown, MichaelNakayama, MizuhoBrownlee, Iain A.Nallasamy, PalanisamyBruggemann, HolgerNanduri, RavikanthBucciantini, MonicaNapoli, PietroBuchler, TomasNapolitano, AlessandraBucki, RobertNarayanaswami, VasanthyBurgalassi, SusiNarod, Steven A.Burger, Michael C.Nasierowska-Guttmejer, AnnaBurnier, JuliaNavarro, EstanisBurns, KatherineNawaz, MuhammadBurrello, JacopoNchinda, Godwin W.Busardò, Francesco PaoloNechepurenko, Ivan V.Businaro, RitaNees, MatthiasBychkov, Maxim L.Negroiu, GabrielaBystrowska, BeataNestler, UlfCaccamo, DanielaNethi, Susheel KumarCademartiri, FilippoNettelbeck, DirkCakstina, IneseNeubauer, HeidiCalabrese, VittorioNeuman, ManuelaCalcagno, SimoneNeunert, GrazynaCaloca, Jose MariaNeureiter, DanielCalvo, PilarNeves, Maria Graça P. M. S.Camejo, GermánNevoral, JanCameron, DonaldNgezahayo, AnacletCampiche, RemoNguewa, PaulCampos, Camila D. MadeiraNguyen, Anh DucCancian, MauroNielsen, Vance GirardCandiota, AnaNieminen, Taina T.Canetta, ElisabettaNieoczym, DorotaCao, Jay J.Nikolova, IrinaCaparrós-González, Rafael ArcángelNilsson, AkeCappellano, GiuseppeNirala, Niraj K.Cappello, PaolaNishimoto, KoshiroCapuano, AlessandroNishioka, HiroshiCapurso, GabrieleNiu, FangCarazo, AlejandroNoain, DanielaCarberry, PatrickNociari, MarceloCardoso, SusanaNoël, AlexandraCarnevale, LorenzoNoguera, Nélida InésCarradori, SimoneNoorani, ImranCarreras, JoaquimNorian, LyseCarugno, JoseNorton, CharlesCasanova, MagaliNota, AlessandroCassim, ShamirNotas, GeorgeCastellano, ImmacolataNwabudike, Lawrence ChukwudiCastellanos, AinaraO’Connor, MichaelCastillo, Carlos A.O’Donovan, SineadCava, ClaudiaO’Gorman, DavidCecchet, FrancescaO’Leary, SeánCecchetti, SerenaOgawa, TomohisaČerná, MarieOgunjimi, Abayomi T.Černáková, LuciaOh, Ji-HyeonCervera, AnaOhashi, KentaroCervetto, ChiaraOhba, ShigeoCha, MyeounghoonOhshima, MitsuhiroChaaban, HalaOkada, TomoyoChachques, Juan CarlosOkiyoneda, TsukasaChacin-Suarez, AudryOku, YoshitakaChan, Koon-HoOkumura, NobuoChang, Chia-ChenOliveira, HélderChang, John C.Oliveira, JorgeChang, Yu-ChaoOlsen, BirgitteChao, CeciliaOmar, Syed HarisCharline, KiefferOng, Peck Y.Chatterjee, SudiptaOperto, FrancescaChauhan, BhareshOpländer, ChristianChavez-Calvillo, GabrielaOrdoñez, LaraChen, Chieh-FuOrekhov, Alexander N.Chen, Chin-ChuanOrellana, RenanChen, Chung-HwanOriente, FrancescoChen, Chun-HanOrsini, MassimilianoChen, LianminOrsucci, DanieleChen, LinOrtiz-Masiá, DoloresChen, QiOrzechowski, ArkadiuszChen, TaipingOsborne, NickChen, XingchengOsna, NataliaCheng, JiaOster, HenrikCheng, JosephOstuni, MarianoCheng, Juei-TangOswald, StefanCheng, Yuan-BinOteri, GiacomoChereddy, KiranOtero-Espinar, Francisco JavierCheret, JérémyOthman, Eman M.Chhabra, GaganOtręba, MichałChiara, María-DoloresOtsuki, TakemiChieppa, MarcelloOuellette, Rodney J.Chintala, SreenivasuluOzen, MehmetChirumbolo, SalvatorePacia, Marta Z.Chiumello, DavidePadula, AndrewCho, Jin AhPahar, BapiCho, Mi HyeonPal, KrishnenduChojnowska, SylwiaPałka, KrzysztofChow, JamesPallag, AnnamáriaChowdhury, IndrajitPalmieri, ErikaChrist, BrunoPalumbo, Vincenzo DavideChrysanthopoulou, AkriviPandha, HardevChung, Seung WooPanos, Anthony L.Chwastek, JakubPantham, PriyadarshiniCianciulli, AntoniaPao, Ping-ChiehCicciù, MarcoPaolmino, OlgaCiccodicola, AlfredoPaone, GaetanoCicero, ArrigoPaplińska-Goryca, MagdalenaCieslar, GrzegorzParadowska-Gorycka, AgnieszkaCignarella, AndreaParanjape, AnuragCinque, BenedettaParaskeva, EfrosyniCiobica, AlinParente, EugenioCione, ErikaParenti, IlariaCipak, LubosParimon, TanyalakClassen, Carl FriedrichPark, Edwards A.Clement, CristinaPark, Eun-JooClemente-Suárez, Vicente JavierPark, Ki SooClementi, MarcoPark, Sae WoongCocchia, NatasciaPark, Shin-HyungCoelho, TracyParkar, Shanthi G.Colasanti, RobertoParlog, RalucaColosimo, CarloParsons, RichardConstantinos, PapagiannitsisPassamonti, SabinaCook, KristinaPatel, Dhaval T.Cooper, KarenPatel, HardikkumarCoppedè, FabioPaton, Madison C. B.Coppo, LuciaPavlik, Edward J.Cordaro, MassimoPawlikowski, MarekCordera, RenzoPedone, ElisaCorrales-Serrano, MarioPeinado, María ÁngelesCosmi, BenildePeiris, DinithiCostabile, AdelePekkala, SatuCosta-Pinto, Ana RitaPellegrino, SaraCourtois, GillesPellis, Sergio M.Courtoy, Guillaume E.Penas, Sonia EirasCouture, RéjeanPenno, MeganCoward, JermainePeraldo-Neia, CaterinaCoyle, Patricia K.Pérez De Vega, María JesúsCrawford, Bryan D.Pérez, María AngelesCremona, OttavioPérez-Gálvez, AntonioCrivii, CarmenPergolizzi, SimonaCruz, CeliaPerše, MartinaCsepany, TundePersechino, SeverinoCurtis, MarionPetralia, Maria CristinaCutone, AntimoPetrov, Alexey M.Cuypers, BartPetta, IoannaCzarnecka, AnnaPhan, Thi Tuong VyCzystowska-Kuzmicz, MalgorzataPiazza, FabrizioCzyż, JarosławPiccinin, ElenaD’Agnano, IgeaPienar, CorinaD’Amico, CesarePievani, AliceD’Angelo, MichelePileggi, ChantalD’Apuzzo, FabriziaPinart, ElisabethD’Argenio, ValeriaPindyurin, Alexey V.D’Orsi, BeatricePinkas, AdiD’Uscio, Livius V.Pipino, CaterinaDa Silva, AnaPiras, CarmenDachineni, RakeshPirklbauer, MarkusDai, ZhiyuPirola, LucianoDal Col, JessicaPiscopo, MarinaDaly, NorellePizzolanti, GiuseppeDamiani, DanielaPizzuti, AntonioDamjanovski, SashkoPlant, LeighDandekar, AdityaPlaza-Díaz, JulioDario, BrunettiPo, AgneseDas, FalguniPoddighe, DimitriDas, PradipPoggi, AlessandroDas, SrustidharPohlkamp, TheresaDas, UndurtiPóka, RóbertDasgupta, PiyaliPolakowski, Nicholas J.Datta, Anita N.Polčic, PeterDau, MichaelPoldermans, DonDaza-Navarro, PaulaPolesello, CédricDe Angelis, BarbaraPolimeno, LorenzoDe Grooth, JorisPoluektova, LarisaDe Jong, StevenPolz-Dacewicz, MałgorzataDe Lellis, LauraPop, CristinaDe Luca, AngelaPopov, AntonDear, Anthony E.Popugaeva, ElenaDeb, SubrataPorcheri, CristinaDegens, HansPorpora, Maria GraziaDel Giudice, RitaPortella, GiuseppeDelle Monache, SimonaPospíšilová, ŠárkaDempsey, Christopher E.Potaczek, Daniel P.Depalma, Ralph G.Poteser, MichaelDesai, DhimantPourbaghi Masouleh, MiladDeschner, JamesPoveda Larrosa, Jose AntonioDevarajan, PriyadharshiniPozzobon, MichelaDevy, JeromePrabhu, LakshmiDey, Amit KumarPrada, IlariaDi Agostino, SilviaPrasad, AnkushDi Baldassarre, AngelaPrasad, VikramDi Domizio, JeremyPriyadarshini, MedhaDi Donato, MarziaProdromidou, AnastasiaDi Martino, OrsolaPucci, MarziaDi Micco, PierpaoloPucéat, MichelDi Resta, ChiaraPuchkov, EvgenyDi Zazzo, ErikaPuglisi, FabioDi, LiPuig, BertaDiaconu, CameliaPujadas, GerardDigiacomo, MariaPushchina, EvgeniyaDimitriadis, StavrosPuthanveetil, PrasanthDimmock, Jonathan R.Puukila, StephanieDinh, Dzung H.Qiu, ZhenDirks, Wilhelm G.Qu, BingqianDivino-Filho, JosèQuigley, RaymondDocea, Anca OanaRabanal Ruiz, YoanaDoddapattar, PrakashRabenstein, Dallas L.Doglietto, FrancescoRagavan, MukundanDogra, PranayRagno, RinoDonnini, SandraRahimi, FaridDorfman, RuslanRahman, Masmudur M.Doroftei, BogdanRahman, Mohammad MijanurDoucet, Gaelle E.Raimondo, DiegoDrougard, AnneRaingeaud, JoëlDroździk, MarekRajendran, Jagathesh ChandraDrutovic, DavidRajendran, VazhaikkurichiDrużyńska, BeataRajpurohit, SurendraDrzeżdżon, JoannaRamchandani, DivyaDuarte, Filipe V.Ramírez, ManuelDudas, JozsefRampazzo, ElenaDudek, JanRanjan, AmareshDufour, AntoineRanjan, AtulDunlop, ElaineRanjan, KishuDupré, Denis J.Ranzato, EliaDupuy, Adam J.Rashid, Mohammad HarunDuquette, PierreRashid, Omar M.Durán-Riveroll, LorenaRastija, VesnaDutta, RajeshRathbone, Alex J.Düzgünes, NejatRatiu, AndreeaDziurkowska, EwelinaRau, IleanaDzobo, KevinRaudenská, MartinaEarl, JulieRauk, ArviEbani, Valentina VirginiaRavaioli, StefanoEchigoya, YusukeRavegnini, GloriaEckschlager, TomasRavuri, SudheerEdwalds-Gilbert, GretchenRecasens, AriadnaEdwards, JoshuaRedina, OlgaEgana Gorrono, LanderRedmer, TorbenEgawa, NagayasuRedondo, Pedro CosmeEid, Ali HusseinReed, Tanea T.Eiró, NoemíRegazzi, MarioEl-Aarag, BishoyRehman, MichaelElango, JeevithanReich, AdamEldridge, SandyReikvam, HåkonEleftheriadis, TheodorosReinhardt, ChristophElida Nora, FerriReis, HenningEl-Khoury, VictoriaRejinold, SanojElla, BhagyarajRekha, Rokeya SultanaEmons, GünterRelat, JoanaEngeland, Christine E.Remaley, AlanEnrico, Gherlone FeliceRemenyik, JuditEnyedy, Éva AnnaRemigante, AlessiaEppard, ElisabethRen, YulinErdélyi, MiklósRenò, FilippoErmolaeva, SvetlanaResheq, Yazid J.Errington-Mais, FionaResiere, DaborEscobedo Monge, Marlene FabiolaRety, StephaneEscoté, XavierReznik, Jacqueline E.Esteve-Romero, JosepRhee, HandooEsworthy, Robert StevenRicci, GiuseppeEverbach, Erich CarrRiccio, PaoloFailla, Cristina M.Richards, Stephen J.Faísca, Patrícia F. N.Richtera, LukášFakhouri, Walid D.Riedel, FabianFalcone, ItaliaRieger-Christ, Kimberly M.Fang, Evandro F.Rigalli, Juan PabloFang, XingRimer, MendellFanti, AlessandroRimpelová, SilvieFarini, AndreaRinninella, EmanueleFarolfi, AndreaRipari, ValeryFatkhudinov, TimurRivera, VictorFavier, Anne-LaureRo, HyunjuFeferman, TaliRoana, JaniraFeng, Hua-JunRoberts, TaraFeng, WanjuanRobson, Matthew JamesFeo, FrancescoRocca, RobertaFerguson, DanielRocha, SoniaFerji, KhalidRocha-Rodrigues, SílviaFernandes, Maria José Da SilvaRoderburg, ChristophFernandez-Abascal, JesusRodilla, VeronicaFernando, I. P. ShanuraRodrigo, LuisFerra, BartłomiejRodrigues, FranciscaFerrara, MicheleRodrigues, Márcia T.Ferrari, EnricoRodríguez, Armando AlexeiFerraro, DianaRoelink, HenkFerreira, SamiraRoger, JérômeFerrigno, AndreaRohrer, BärbelFerro, RiccardoRojo, Maria AngelesFilipek, AgnieszkaRomanazzo, SaraFinol-Urdaneta, Rocio K.Romani, AndreaFischer, AndreasRomano, LorenzoFischer, Michael B.Rosales, CorinaFisicaro, FrancescoRoseanu, AncaFlaatten, HansRoshandel, DelnazFoecking, MelanieRossi, FrancaFoglio, EleonoraRossi, FrancescaFontana, Barbara D.Rothschild, BruceFonte, Maria LuisaRoue, GaelFord, JonRouthu, Nanda KishoreForest, FabienRoy, Saktimayee MitraForlano, RobertaRóżańska, AnnaFormanowicz, DorotaRubiano, AndresFormisano, PietroRucinski, MarcinFornasaro, StefanoRuiz, MarioFortunato, LeonzioRuiz, MatthieuFrancés-Soriano, LauraRumney, PeterFranco, IreneRund, DeborahFranco, RodrigoRuoss, MarcFrançois, CasasRusanova, IrynaFrank, SašaRusso Krauss, IreneFrazzi, RaffaeleRusu, Laura CristinaFreidl, WolfgangRutzler, MichaelFry, Bryan GriegRuysschaert, Jean-MarieFu, LianwuRybska, MartaFu, Shu-LingRybtsov, StanislavFuchs, OtaRyll, BettinaFujimori, KoRyo, UshiodaFujioka-Kobayashi, MasakoRyugo, DavidFujita, MitsuguRzymowska, JolantaFukui, TomoyasuSá, RosáliaFuruta, TakuyaSabatino, LauraFushida, SachioSaccà, Sergio ClaudioGacesa, RankoSachio, TsuchidaGafencu, AncaSachithanandham, JaiprasathGagniuc, PaulSacitharan, Pradeep KumarGall Troselj, KoraljkaSadler, AnthonyGallego, Óscar S.Sahani, RajkumarGalley, Helen F.Sahuquillo, JuanGalochkina, Tatiana V.Sakai, AtsushiGaman, Mihnea-AlexandruSakata, NaoakiGan, SamuelSaler, MarcoGandhirajan, Rajesh KumarSalto-Gonzalez, RafaelGandini, PaolaSamaja, MicheleGanguly, AvishekSamartzis, Eleftherios PierreGaranto, AlexSamir, ParimalGarcia, SamuelSamykutty, AbhilashGarcía-Barrado, María JoséSanchez Vargas, Luis AlbertoGarcía-Díaz, MaríaSanchez, AnaGarcia-Gonzalez, NataliaSánchez-Fidalgo, SusanaGarcía-Otín, Ángel LuisSancho-Vaello, EneaGarcia-Pardo, JavierSands, Jeff M.Garcia-Sosa, AlfonsoSanna, FabrizioGarcia-Verdugo, IgnacioSantabarbara, JavierGarcía-Vicente, Ana MaríaSantacroce, LuigiGarofalo, MariangelaSantarelli, AndreaGarozzo, DomenicoSantos, Fátima MilhanoGarriga, PereSantulli, GaetanoGarzon, SimoneSanz, CarmenGashti, Mazeyar ParvinzadehSapozhnikov, Alexander M.Gasperi, TeclaSapudom, JiranuwatGauthier, BenoitSaravanakumar, KandasamyGazzin, SilviaSardeli, ChrysanthiGe, XiaodongSarkozy, ClémentineGe, XinSarma, SauravGelzo, MonicaSarmah, SwapnaleeGempt, JensSato, DaisukeGenazzani, Alessandro D.Sato, EiichiGendaszewska-Darmach, EdytaSato, ShuzoGennai, StefanoSato-Bigbee, CarmenGenovese, CarloSaturnino, CarmelaGentile, FrancescoSauer, AchimGentilucci, LucaSavino, FrancescoGeorgakilas, AlexandrosSawada, AkinariGeorgescu, AdrianaScafoglio, ClaudioGeorgiev, VasilSchäfer, KatrinGerber, Leonard E.Schiavone, NicolaGerding, HeinrichSchilling, Arndt FriedrichGerke, OkeSchirhagl, RomanaGermana, AntoninoSchmidt, JustinGessler, FlorianSchmoll, DieterGessner, GuidoSchneider, JochenGeuns, Jan M.C.Schrezenmeir, JuergenGhosh, ArnabSchrier, RachelGhosh, SantoshSchrum, AdamGiacomazza, DanielaSchubert, UlrichGiacomelli, ChiaraSchulze, JohannesGiammanco, AntoninaSciacca, Michele Francesco MariaGiannoni, PatriziaScicchitano, PietroGiganti, FrancescoScioscia, CrescenzioGil, KrzysztofScotto D’Abusco, AnnaGillespie, James W.Sebastian, AimyGillet, GermainSeene, TeetGillet, Jean PierreSeidelman, William E.Ginzburg, Yelena Z.Seiler, Magdalene J.Giordano, AntonioSelleri, SilviaGiralt, AlbertSenbonmatsu, TakaakiGiralt, ErnestSerafini, GianlucaGiri, HemantSerefko, AnnaGiulia, MauriziSessa, FrancescoGiussani, PaolaSgrignani, JacopoGiusti, IlariaShabbir, WaheedGłąbska, DominikaShahi, Shailesh K.Gladkikh, IrinaShang, QingsenGlasscock, EdwardSharma, NaveenGlazar, IrenaSharma, PreetiGlushakov, AlexanderSharma, SudhaGmeiner, William H.Shaul, Yoav D.Goenka, ShilpiSheng, YueGolan, DanielShenoy, Anukul T.Golay, JoseeShete, VarshaGoldsmith, Jason R.Shi, LeiGomes, Pedro S.Shigemura, YasutakaGomez-Llorente, CarolinaShih, Jing-WenGoni, FelixShimada, YohtaGonzález-Scarano, FranciscoShimaoka, MotomuGoodman, Simon L.Shimo, TsuyoshiGöpel, SvenShinde, AparnaGoris, TobiasShinde, Arti V.Gottesman, Michael M.Shinohara, YasuoGoyvaerts, CleoShinoka, ToshiharuGradoni, LuigiShiota, SeijiGraier, WolfgangShiraishi, KenshirouGranados-Principal, SergioShivapurkar, NarayanGrand, RogerShmarakov, IgorGrande, RaffaeleShukla, ArvindGrange, Robert W.Shukla, GirishGratacap, Marie-PierreSiamidi, AngelikiGriffith, Thomas S.Siddappa, ByrareddyGroome, JamesSiegel, Sue RutherfordGrossi, ValentinaSigala, SandraGrotz, Travis E.Sil, PayelGrover, NatalieSilva, Amelia M.Grubić Kezele, TanjaSilva, AnjanaGrywalska, EwelinaSilvestris, NicolaGrzybowska, EwaSimka, MarianGu, ZhenglinSimko, FedorGulic, TamaraSimon, JorgeGullbrand, Sarah E.Singh, AnandGunalan, VithiagaranSingh, ReemaGuo, PuSingh, SarbjitGupta, HimanshuSinha, JasmineGupta, NehalSiniscalco, DarioGupta, SanjaySiracusano, SalvatoreGurevich, Vsevolod V.Sita, GiuliaGuriec, NathalieSkirtach, AndreHaak, HarmSlack, Frank J.Hachiya, NaomiSliwinska, AgnieszkaHa-Duong, Nguyet-ThanhŚliwińska-Mossoń, MariolaHaendler, BernardSłomka, ArturHahn, OliverSmerdou, CristianHajduch, EricŠmída, MichalHałas-Wiśniewska, MartaŚmielak, BeataHalloran, KieranSmith, MiaHalperin, John J.Smith, Micholas DeanHamada, Yahia Z.Smolkova, BozenaHamano, TadanoriSobotta, ŁukaszHammer, ElkeSoica, CodrutaHamułka, JadwigaSolanki, AshishHan, SongSolomonov, InnaHanania, MichelSolyanik, OlgaHandral, HarishSomvanshi, Rishi K.Handzel, OphirSonkin, DmitriyHans, Chetan P.Sonnemann, JürgenHaque, SanjanaSorice, MaurizioHarada-Shiba, MarikoSotgiu, StefanoHarandi, Vahid M.Soueidan, AssemHart, MelanieSoulen, Michael C.Hartman, MariuszSousa, Sérgio F.Hartsough, EdwardSpampinato, SantiHashimoto, YoshitakaSpella, MagdaHatch, Grant M.Splichal, IgorHattori, ToshioSridhar, SubbaramiahHayashi, KazuhikoSrinivas, JanaswamyHayashi, YoheiSrinivasan, BharaniHe, John CijiangSripathy, SmithaHe, LixiaSrivastava, AkritiHellmann, MarcinSrivedavyasasri, RadhakrishnanHelsley, Robert N.Stacewicz-Sapuntzakis, MariaHenstridge, ChristopherStange, KatjaHerdewijn, PietStarčević, AntonioHernandez Fernandez, AntonioStaudacher, AlexanderHerzig, VolkerStecco, CarlaHibbitts, AlanStefania, NiadaHildebrandt, Jan-PeterStefania, RacheleHildebrandt, SabineStefanucci, AzzurraHildreth, Blake E.Stehle, JörgHildreth, EasonStella, Giulia M.Hilhorst, MarcStemmler, MarkHill, DavidStepanova, Anna A.Hiraga, ToruSterk, Geert JanHiraoka, SakikoStewart, AlanHirota, YasushiStobiecka, MagdalenaHirsch, IvanStock, UlrichHisako, YoshidaStockdill, Jennifer L.Hissa, BarbaraStorsberg, JoachimHo, RogerStott, Jennifer B.Hofbauer, StefanŠtrac, Dubravka ŠvobHoffman, JessicaStragliotto, GiuseppeHoffmann, JedrzejStratmann, BerndHohensinner, PhilippStratton, Margaret M.Hokugo, AkishigeStrudwick, XantheHollis, Robert L.Stupka, NicoleHolt, KellySud’ina, Galina F.Homma, TetsuyaSugimoto, NaotoshiHonda, YasushiSujeesh, SebastianHoneychurch, JamieSundaram, Sivaraj MohanaHori, MikaSurguchov, AndreiHorstmann, MarcusSuwan, KeittisakHortells, LuisSuzuki, MasakoHortobágyi, TiborSvobodová, JanaHossler, Eric W.Swami, UmangHrabal, RichardSwierczynski, JulianHsia, Shih-MinSzeto, Yim-TongHsiao, Yi-HsuanTabęcka-Łonczyńska, AnnaHsieh, Wen-TsongTabuchi, MasashiHsu, Bang-GeeTaguchi, YoshihiroHsu, Tsung-ITakahashi, KenHu, JunhuiTakahata, YoshifumiHu, YuanTakano, KatsuraHuang, Fu-ChenTakasawa, ShinHuang, Li-TungTakashi, SekiyaHuang, TimothyTaked, TakayukiHuang, Tse-HungTakegahara, NorikoHuang, WeichenTamanoi, FuyuhikoHuang, YueQiaoTambuwala, MurtazaHuck, OlivierTamm, Katja PokrovskajaHuckriede, AnkeTan, Bee K.Huerta-Angeles, GloriaTan, JoanneHułas-Stasiak, MonikaTan, Shyh HanHuleihel, MahmoudTanaka, HiromiHurwitz, StephanieTanaka, Hiroyoshi Y.Hutzen, BrianTanaka, MasashiIatsunskyi, IgorTanaka, TakujiIdoate, MiguelTanaka, TeruyoshiIhara, SozaburoTang, AnnaIkegaya, NaokiTaniguchi, HiroakiIkura, TeikichiTarantino, GiovanniIlluminati, GiulioTartey, SarangIm, Chang-NimTashima, ToshihikoImpellizzeri, DanielaTata, AlessandraImperatore, RobertaTatullo, MarcoInaba, HiroshiTedesco, IdoloInagaki, HidetoshiTeisseyre, AndrzejInfante, ArantzaTeixeira, LeandroIngelman-Sundberg, MagnusTekriwal, AnandInokuchi, TsutomuTenchov, BorisIommelli, FrancescaTeng, Ba-BieIriepa Canalda, IsabelTeodorescu, FlorinaIriuchishima, HironoTeragawa, HirokiISHIAI, MasamichiTerasaki, MichishigeIshii, KenichiroTerkawi, Mohamad AlaaIskra, MariaTessier, FredericItabe, HiroyukiTesta, UgoIto, ShosukeTestai, LaraIvanov, AlexanderTevosian, Sergei G.Ivask, AngelaThackray, AlanaIwamoto, ShotaroThakor, Avnesh S.Jaglal, Michael V.Thankam, Finosh G.Jahangiri, LeilaThieme, RenéJahroudi, NadiaThomas, Stephen J.Janecka, AnnaThurnher, MartinJang, HyoncholTienari, PenttiJang, Sung-WukTimmerman, PeterJangampalli Adi, PradeepkiranTiriveedhi, VenkataswarupJanikowska, GrażynaTobiasch, EddaJanowski, MiroslawToden, ShusukeJarończyk, MałgorzataTogawa, AtsushiJavaheri, TaherehToillon, Robert-AlainJayant, RahulTolosa, EzequielJayaraman, ShobiniTomkin, Gerald H.Jensen, Rebekka VibjergTonelli, MicheleJho, Hyun JungTong, MingmingJia, ZheTong, XinJiménez-Tenorio, ManuelTopczewski, JacekJin, Byung RaeTorisawa, YusukeJin, ZhuqiuToro, Mario DamianoJohansen, Matt D.Török, PeterJohnson, Colin P.Torre, Maria LuisaJohnstone, TimTorsello, BarbaraJoseph, Justin V.Tósaki, ÁrpádJuif, Pierre EricToth, RekaJung, Hye JinToth, ZsoltJung, Ji HoonToti, KiranJung, Sung ChulTotu, EugeniaJunka, AdamToyoda, MasashiJurcek, OndrejToyokawa, TatsuyaJurkowska, HalinaTraini, ToninoJutzeler, Catherine J.Trant, JohnJyotsna, MishraTrček, JanjaKaaja, RistoTriantafillidis, John K.Kabała-Dzik, AgataTrigatti, BernardoKadela-Tomanek, MonikaTripathi, MadhulikaKaessmeyer, SabineTriposkiadis, FilipposKaida, DaisukeTriunfo, StefaniaKaji, HiroshiTrounson, AlanKalani, KomalTrubiani, OrianaKale, AbhijitTrusca, Georgeta VioletaKalland, Karl-HenningTsai, Chia-LiangKalyuzhnaya, MarinaTsai, Ming-ShaoKamimura, KenyaTsampras, NikolaosKaminski, Tomasz W.Tsigalou, ChristinaKämmerer, Peer W.Tsirigotis, PanagiotisKanaujiya, JitendraTsitsilonis, Ourania E.Kaneko, KazunariTsuchida, SachioKang, DongchulTsukamoto, ShoKantola, Ilkka M.Tsutsumi, HiroshiKaplan, BarbaraTsytsarev, VassiliyKar, AdwitiyaTuccari, GiovanniKaraduta, OlegTurato, CristianKarageorgos, SpyridonTurchetti, DanielaKarapanayiotides, TheodoreTurnbull, JeremyKarapetis, StefanosTurner, JonathanKaravasili, ChristinaTuzun, UgurKarmouty-Quintana, HarryTyakht, AlexanderKarpiński, Tomasz M.Tzeng, Chii-ReuyKaschina, ElenaUddin, Golam MezbahKashchenko, Nina I.Ulasov, IlyaKashiwagi, ShinichiroUllevig, SarahKashtalap, VasilyUm, SoyounKassayová, MonikaUmemori, JuzohKast, Richard E.Umemura, TsukuruKatagiri, KazumotoUpham, Brad L.Katakam, PrasadUranga, JoseKato, HirotomoUrbinati, GiorgiaKatona, EvaUsui, TatsufumiKaushik, Nagendra KumarUyeda, TaroKawahata, IchiroVaandrager, Arie B.Kawakami, JunVaccaro, Maria GraziaKawauchi, KeikoVacher, PierreKeisaku, SatoVafiadaki, ElizabethKejík, ZdeněkVaibhav, KumarKeppler, DanielVaiyapuri, SakthivelKerekes, GyorgyVakrou, StylianiKhaddaj Mallat, RayanValenti, MariaKhambu, BilonValko, MarianKhan, IrfanVallée, AlexandreKhan, SabbirValm, Alex M.Khanna, HemantVan Cruchten, Steven J.Khaw, Kooi YeongVan Den Heuvel, LambertusKhemtong, CharlieVan Etten, Ellis S.Kholová, IvanaVan Hoek, BartKibler, Karen V.Vanzi, FrancescoKijewska, MonikaVáradi, CsabaKikuchi, TaiseiVarela, IgnacioKilari, SreenivasuluVarga, ZoltánKim, BongjuVarghese, MathewsKim, In-BeomVarone, FrancescoKim, JihoonVassaux, GeorgesKim, Keun-SikVassiliou, Aliki GeorgiaKim, Kyoung SooVasto, SonyaKim, KyuseokVazquez, Elba S.Kim, Seok-HoVecchione, Dr RaffaeleKim, SeonghoVecoli, CeciliaKim, SoochongVekaria, HemendraKim, Sung-KyoVeldhuizen, Ruud A. W.Kimura, TadashiVelu, Sadanandan E.Kindy, Mark S.Vemuri, RavichandraKiss, RitaVenkannagari, HarikanthKiss, RobertVenkataraman, ShrinivasKita, ToshihiroVergés, MarcelKitagawa, SeiichiVerri, TizianoKitoh, HiroshiVerzak, ŽeljkoKlar, AgnesVesa, Cosmin MihaiKlein, BrittaVesa, Stefan CristianKloc, MalgorzataVetvicka, VaclavKlughammer, JohannaVíglaský, ViktorKnapczyk-Stwora, KatarzynaVinarov, ZahariKnäuper, VeraVingolo, Enzo MariaKnobloch, KarstenVisco, VincenzoKnudsen, GabrielVisconte, ValeriaKobeissy, FirasViswanathan, SowmyaKodidela, SunithaVlad, Cătălin IoanKoduru, JanardhanVlkova, MarcelaKoek, GerVolk, DavidKoga, YoshikatsuVon Der Thüsen, JanKoh, ChristopherVostinaru, OliviuKoike, HarukiWalenga, Jeanine M.Koivisto, AriWalker, Douglas G.Kojima, ShihokoWalker, SteveKok, WouterWallace, MartinaKolbenschlag, JonasWan, LeiKolekar, PandurangWang, Chih-HungKoli, SwanandWang, Chin-TienKolluru, GopiWang, HaitaoKomosinska-Vassev, KatarzynaWang, Jin’AnKonstantinidis, Theocharis G.Wang, KankanKorbecki, JanWang, PingyuanKordyukova, LarisaWang, PiwenKorivi, MallikharjunWang, ShikeKortaberria, GalderWang, Shin-WeiKosinsky, Robyn LauraWang, Tsung-JenKostner, Gert M.Wang, XiaohongKotula-Balak, MałgorzataWang, XinyuKou, YiWang, ZhijunKoutelidakis, Antonios E.Want, Muzamil YaqubKovalenko, Elena I.Ward, MicaiahKowol, Christian R.Ward, Roberta J.Koya, DaisukeWatała, CezaryKozakiewicz, AnnaWaters, PatrickKozlov, SergeyWautier, Marie-PauleKrag, Thomas. O.Wawruszak, AnnaKrajnak, Kristine M.Weaver, JolantaKrauklis, Andrey E.Weber, David S.Krebs, MarkusWeber, ViktoriaKrick, StefanieWeighardt, HeikeKruszelnicka, OlgaWenzl, RenéKrzystanek, MarekWesołowska, OlgaKshatri, AravindWesołowski, MarekKuan, Yu-HsiangWeyd, HeikoKugelman, Jeffrey R.Wiggs, MichaelKulandavelu, ShathiyahWilhelm, KerstinKulkarni, Krishnaji R.Williams, Noelle S.Kumar, AvneeshWilm, MatthiasKumar, ShaileshWilson Pastuck, TawnyaKumar, UjendraWilson, DavidKumar, VijayWilson, Jamie L.Kummer, KaiWingert, RebeccaKunz, WolframWinter, AlexanderKuo, Ming-TseWinter, ElizabethKurakula, MalleshWiraja, ChristianKushima, MikiWittmann, MiriamKutikhin, Anton G.Wlaź, PiotrKvitek, LiborWnuk, MaciejL’homme, LaurentWohlleber, DirkLa Rosa, PiergiorgioWojtukiewicz, MarekLaboisse, Christian L.Woo, So-YounLabrou, NikolaosWoods, Erik J.Lach, SławomirWozniak, MichalLachowicz, Joanna IzabelaWrobel, MariaLacina, LukasWu, AlanLadekarl, MortenWu, ChuanjinLaganà, Antonio SimoneWu, Chung-ChunLahiri, SatadruWu, Chung-HsinLai, MeimeiWu, ChunsenLai, Michael M. C.Wu, JenderLakey, Jonathan Robert ToddWu, Jia PingLakshminarayanan, RajamaniWu, NanLalli, EnzoWu, Suh-ChinLampiasi, NadiaWu, XianfangLancelin, Jean-MarcXiaobin, HuangLanza, GiuseppeXiong, Wen-ChengLaranjo, MafaldaXu, JianLatsios, GeorgeXu, JinbinLau, Eric K.Xue, XufengLaura, EvangelistaYadav, DhananjayLauro, DavideYadav, Dharmendra K.Lavarino, CinziaYadav, TribhuwanLaw, BenedictYager, DorneLawson, KimYallapu, MuraliLay, Horng-LiangYamada, HiroyukiLayer, Paul G.Yamada, ShuheiLazarević, VladimirYamanaka, KeiichiLazzara, GiuseppeYamato, MasayukiLeach, KatieYan, Y. SandersLecis, DanieleYang, BoLee, Dong YunYang, ChunzhangLee, Gun WooYang, JiangningLee, Hwa-JinYang, JunhuaLee, Meng-JenYano, YoshihikoLee, SangheeYarom, NoamLee, SeokHeeYarowsky, PaulLee, Seung HeeYasuhara, KazumaLee, SukmookYee, NelsonLee, Tzong-ShyuanYeh, Chau-TingLee, Yu-HsuanYen, Yi-KuangLegaz, IsabelYi, Hee-GyeongLei, WeiYokohira, MasanaoLei, ZhentianYokoi, TsuyoshiLeigh, JamesYoshida, KatsunoriLeiphrakpam, PremilaYoshihara, ToshinoriLemli, BeátaYoshikazu, NakamuraLentini, GiovanniYoshino, OsamuLeo, Alfredo DiYoshitake, HiroshiLeonard, FransiscaYousefi, HassanLeonardi, AdrijanaYousefi, ShidaLesauskaitė, VitaYu, JunweiLetek, MichalYumura, YasushiLevesque, EricYura, YoshiakiLewandowska-Szumieł, MałgorzataYzydorczyk, CatherineLewis, AnnabelleZaboronok, AlexanderLi, Chia-JungZacchia, MiriamLi, WeijuanZádor, FerencLi, Wen-WuZaffaroni, NadiaLiagre, BertrandZaiss, DietmarLiakina, ValentinaZambelli, FilippoLian, IanZamboni, PaoloLiang, Shun-QingZamorano-Leon, José JavierLiao, WupengZangerl, GerhardLiao, ZehuanZapico, Sara C.Liapis, GeorgeZaravinos, ApostolosLiktor-Busa, ErikaZarogoulidis, PaulLim, Ki-TaekZarrelli, ArmandoLin, AthenaZavattaro, ElisaLin, WeifengZbigniew, BrzozkaLindemann, StephanZdrowowicz, MagdalenaLindstedt, SandraZdzisińska, BarbaraLiontos, MichalisZeleny, ReinhardLipińska, AndreaZhai, KuiLisi, LuciaZhang, GuofangLisowska, KatarzynaZhang, LiqiangLita, AdrianZhang, ShuangLitak, JakubZhang, YuLitwin, TomaszZhang, ZhibingLiu, Chao-LinZhao, GexinLiu, Cheuk LunZhao, LinboLiu, HengruiZhao, QingnanLiu, Jun-JenZhao, YueLiu, XiaoyingZheng, GuopingLizio, GiuseppeZheng, QiLo Monte, Attilio IgnazioZheng, Yun-WenLo, Leu-WeiZhou, ZhijunLocatelli, MarcelloZhu, YuLode, AnjaZhuang, XiaoxuanLonardo, AmedeoZiemnicka, KatarzynaLoosli, FelixZimta, Alina-AndreeaLópez, SergioŻołądek, TeresaLopresti, PatriziaZollner, ThomasLordan, RonanZubair, HaseebLorenzo-González, ÓscarZumerle, SaraLosurdo, GiuseppeZurita, MarioLu, Hong S.


